# Mechanical Properties and Corrosion Rate of ZnAg3 as a Novel Bioabsorbable Material for Osteosynthesis

**DOI:** 10.3390/jfb15020028

**Published:** 2024-01-25

**Authors:** Maria Roesner, Sergej Zankovic, Adalbert Kovacs, Moritz Benner, Roland Barkhoff, Michael Seidenstuecker

**Affiliations:** 1 G.E.R.N. Tissue Replacement, Regeneration & Neogenesis, Department of Orthopedics and Trauma Surgery, Medical Center-Albert-Ludwigs-University of Freiburg, Faculty of Medicine, Albert-Ludwigs-University of Freiburg, Hugstetter Straße 55, 79106 Freiburg, Germany; maria.roesner@uniklinik-freiburg.de (M.R.); sergej.zankovic@uniklinik-freiburg.de (S.Z.); 2 Limedion GmbH, Coatings and Surface Analysis, Am Schäferstock 2-4, 68163 Mannheim, Germany; kovacs@limedion.de (A.K.); m.benner@limedion.de (M.B.); 3 Quadralux e.K., Am Schäferstock 2-4, 68163 Mannheim, Germany; r.barkhoff@quadralux.de

**Keywords:** zinc silver alloy, corrosion rate, shear testing, tensile testing, osteosynthesis, biodegradable

## Abstract

Osteosynthesis in fracture treatment typically uses hardware that remains in the patient’s body, which brings a permanent risk of negative side effects such as foreign body reactions or chronic inflammation. Bioabsorbable materials, however, can degrade and slowly be replaced by autologous bone tissue. A suitable material is requested to offer great biocompatibility alongside excellent mechanical properties and a reasonable corrosion rate. Zinc–silver alloys provide these characteristics, which makes them a promising candidate for research. This study investigated the aptitude as a bioabsorbable implant of a novel zinc–silver alloy containing 3.3 wt% silver (ZnAg3). Here, the tensile strength as well as the corrosion rate in PBS solution (phosphate buffered solution) of ZnAg3 were assessed. Furthermore, shear tests, including fatigue and quasi-static testing, were conducted with ZnAg3 and magnesium pins (MAGNEZIX^®^, Syntellix AG, Hannover, Germany), which are already in clinical use. The detected corrosion rate of 0.10 mm/year for ZnAg3 was within the proposed range for bioabsorbable implants. With a tensile strength of 237.5 ± 2.12 MPa and a shear strength of 144.8 ± 13.2 N, ZnAg3 satisfied the mechanical requirements for bioabsorbable implants. The fatigue testing did not show any significant difference between ZnAg3 and magnesium pins, whereas both materials withstood the cyclic loading. Thus, the results support the assumption that ZnAg3 is qualified for further investigation.

## 1. Introduction

In orthopedics and trauma surgery, many fractures are treated surgically by using hardware such as screws and nails. To ensure a sufficient healing process, the implanted material should offer the needed stability as well as a distinct biocompatibility. Nonetheless, many materials remain inside the body since they do not degrade, which can lead to serious foreign body reactions and inflammation, and consequently might require implant removal [1,2,3,4,5,6]. By developing bioabsorbable materials, this issue is addressed in the scientific community. Here, the human body can regain a fully healed bone structure consisting of autologous tissue after a fracture by absorbing the implanted material.

Currently, only polymer-based and magnesium-based materials are in clinical use [4,7]. Although materials such as polyglycolic acids and polylactic acids might show mostly satisfying results in special fields such as the treatment of pediatric maxillofacial fractures, several limitations have occurred, such as poor tensile strength and foreign body reactions with local swelling, sterile abscess, and fistula formation [8,9,10]. Since magnesium and its alloys reach the targeted tensile strength for bioabsorbable materials of 200 to 300 MPa, they have been attracting attention in this research field [11,12,13]. However, the high corrosion rate of magnesium at 0.2–2 mm/year and the formation of hydrogen gas pockets inside the tissue are remaining concerns in clinical use [14,15,16]. A corrosion rate that is too high can lead to premature degradation of the implant material and thus does not guarantee sufficient mechanical stability for the bone during the healing process. Furthermore, the degradation of magnesium leads to an increase in pH value within the implant environment, which has negative effects on the surrounding tissue [4]. This can disrupt the healing process and thus delay bone reconstruction [17].

Zinc alloys provide distinguished biocompatibility and meet the requested mechanical parameters, which is why they came to the attention of researchers [18,19,20,21,22,23]. Zinc is one of the trace elements of the human body and is involved in many vital processes [24]. Among other things, zinc is known to have crucial effects on bone formation by activating aminoacyl-tRNA synthetase in osteoblastic cells and thus increasing bone formation [25]. It is further known that zinc disrupts bone resorption by interfering with osteoclast-like cell formations [24,25]. Zinc also affects mesenchymal stem cells by increasing their growth rate and influencing their cell survival positively [26]. Furthermore, Ma et al. [27] showed that zinc supports cell migration and cell proliferation in smooth muscle cells. However, pure zinc does not offer the necessary mechanical stability, which is why it has to be alloyed with other metals such as silver to reach the benchmarks of the proposed mechanical properties for bioabsorbable materials [18,28]. By alloying zinc with silver, the ultimate tensile strength (UTS) can be increased up to 287 MPa, compared to 100–150 MPa for pure zinc [29,30].

Silver has been intensively researched as an implant material and has shown excellent biocompatibility [31,32]. It is proven that silver supports bone regeneration in the cancellous bone and, therefore, is a promising choice as an implant material [31]. Here, Zhang et al. [33] also showed that silver improves the healing process after fracture by introducing silver nanoparticles. Here, silver would increase the mesenchymal stem cell proliferation in the mouse model. Moreover, silver exhibits antibacterial properties by preventing the adhesion of bacteria to the surface of the material [34]. This material property is intensively researched in several medical fields to prevent bacterial infections after implantation [35,36,37].

Studies showed that the silver content of zinc–silver alloys correlates with the enhancement of the mechanical properties and, furthermore, alters the corrosion rate as well as the biocompatibility [30,38]. Zinc–silver alloys with an Ag percentage of, e.g., 6 wt% showed a UTS of 211 MPa, a yield strength (YS) of 213 MPa, and an elongation at fracture of 25% compared to pure zinc with a UTS of 115 MPa, a YS of 110 MPa, and an elongation at fracture of 9.7% [38]. In addition, the grain size has an enormous impact on the mechanical properties. Metals with smaller grains can have greater ductility, which is important for ensuring adequate osteosynthesis [39]. A moderate elongation at fracture is one key to a successful implant material. Hence, for orthopedic internal fixation devices, an elongation at fracture of 20–40% is recommended [40].

The degradation rate of zinc with a standard electrode potential of −0.76 V is lower and more beneficial for bone healing than that of magnesium with a standard electrode potential of −2.38 V [41,42]. The corrosion rate of zinc–silver alloys increases the higher the Ag content is and lies approximately between 0.05 and 0.15 mm/year [38]. This is within the suggested range of 0.04–0.14 mm/year for bioabsorbable implants in orthopedics and trauma surgery [18]. The degradation of zinc also does not lead to the formation of hydrogen gas pockets, as is the case for magnesium [14,43,44,45]. During the degradation process, the material slowly becomes absorbed by the patient’s body. Here, Vojtěch et al. [19] could display that the amount of zinc exposed to the metabolism is harmless and does not cause any negative effects since it is far below the tolerable daily dose of zinc with a limited maximum intake of 40 mg/day [19,46].

In this study, the mechanical properties and corrosion rate of ZnAg3 were investigated. The objective of this study was the qualification of this novel alloy with regard to its mechanical properties in order to validate its suitability for clinical use. The alloy examined has not yet been described in the literature with regard to its mechanical properties and may represent a novel material for osteosynthesis. The zinc–silver alloy contained 3.3 wt% Ag and showed promising biocompatibility on human osteoblasts in previous investigations, which is why this alloy was selected for further investigations [47]. More details about the tested alloy can be found in a previously published paper characterizing the alloy [47]. The alloy in this study was not coated and did not receive any surface modifications. To assess its aptitude as a bioabsorbable implant for osteosynthesis, mechanical measurements were conducted and included tensile tests and shear tests. For the shear tests, the ZnAg3 pins manufactured by Limedion and Quadralux (Limedion GmbH, Mannheim, Germany; Quadralux e.K., Mannheim, Germany) were compared to magnesium pins (MAGNEZIX^®^, Syntellix AG, Hannover, Germany), which are already in clinical use. Here, the samples were subjected to typical loads after hallux-valgus surgery to examine the fatigue strength [48]. The quasi-static strength was assessed by loading the samples to the point of failure. Moreover, corrosion measurements were conducted to confirm the supposed degradation rate within the proposed range for bioabsorbable implants.

## 2. Materials and Methods

### 2.1. Tensile Testing

The tensile tests were conducted by the State Materials Testing Institute Darmstadt, Germany, where dog bone-shaped specimens of ZnAg3 manufactured by Limedion and Quadralux (Limedion GmbH, Mannheim, Germany; Quadralux e.K., Mannheim, Germany) were tested according to ISO standard 6892-1:2020-06 [49]. The samples measured 4 mm in diameter and 20 mm in length. Measurements were conducted at room temperature with a class 1 universal testing machine (Hegewald & Peschke Inspect 250, Hegewald & Peschke Mess- und Prüftechnik GmbH, Nossen, Germany). Tensile strength and yield strength were investigated for ZnAg3. Elongation at fracture for ZnAg3 was determined according to ISO standard 6892-1:2020-06 Appendix I (Determination of elongation at fracture at subdivision of the initial measuring length) [49].

### 2.2. Shear Testing

To assess the mechanical performance, shear tests were performed with ZnAg3 pins manufactured by Limedion and Quadralux (Limedion GmbH, Mannheim, Germany; Quadralux e.K., Mannheim, Germany) with a diameter of 2 mm and a length of 30 mm. For comparison, MAGNEZIX^®^ pins (Serial No. 1127.030, Syntellix AG, Hannover, Germany) with the same diameter and length were also tested. To simulate physiological conditions, the pins were inserted into 20 PCF Cellular Rigid Polyurethane Foam (1522-12, Sawbones USA, Pacific Research Laboratories Inc., Vashon, WA, USA) as a functional bone substitute material. After predrilling the blocks with a standing drill (BF M3, Arnz Flott GmbH, Remscheid, Germany) at 650 rpm, a pin was inserted using an impactor (Impactor 6127.010, Syntellix AG, Hannover, Germany). Two blocks were joined together with a pin and deflected against each other during the shear tests.

Quasi-static strength and fatigue strength were evaluated by performing generic loading scenarios comparable to general loading conditions after hallux-valgus surgery. All mechanical measurements were conducted with a servo-hydraulic testing machine (Amsler HC 10, Zwick-Roell GmbH, Ulm, Germany) equipped with a 10 kN load cell as well as an eligible software (TestXpert R V1.4.2, Zwick-Roell GmbH, Ulm, Germany).

#### 2.2.1. Fatigue Testing

A force-controlled, sinusoidal dynamic loading at 3 Hz for 250,000 cycles was applied to the pins to simulate the average walking load after hallux-valgus surgery [48]. The fatigue testing was conducted with a preload of 18 N and was cyclically loaded with a maximum force of 20 N or 30 N, and a minimum force of 6 N, which corresponds to a loading period of approximately 6 weeks assuming 5000–7000 cycles per day [48]. The recording was made with 1000 values per second for the applied force (N) and deformation (mm). A displacement of >2 mm was defined as pin failure [50,51].

#### 2.2.2. Quasi-Static Testing

The pins were loaded to the point of failure, defined as a deformation of >2 mm, in displacement-controlled static tests at a rate of 5 mm/min. The recording was made with 1000 values per second for the applied force (N) and displacement (mm). Thus, the ultimate load (F_max_) could be determined.

### 2.3. Electrochemical Testing

To determine the corrosion rate, test specimens (Limedion GmbH, Mannheim, Germany; Quadralux e.K., Mannheim, Germany) with a diameter of 6 mm, a height of 1 mm, and an exposed surface area of 0.2827 cm^2^ were secured watertight in a sample holder made of FC53 polyol and FC52/23 isocyanate (Rencast^®^, OBO-Werke GmbH, Stadthagen, Germany). The samples were then placed in a corrosion measuring cell (KMZ5, Sensortechnik Meinsberg, Xylem Analytics Germany Sales GmbH & Co. KG, Waldheim, Germany), which was filled with PBS solution (8 g/L NaCl, 0.2 g/L KCl, 1.44 g/L Na_2_HPO_4_, 0.245 g/L KH_2_PO_4_) and equipped with a potentiostat/galvanostat PS2000, an Ag/AgCl reference electrode SE11, and a counter electrode made of a 4 cm^2^ Pt foil. The measurements were conducted at a pH of 7.4 ± 0.2 and a temperature of 37 ± 2 °C.

At the beginning of the measurements, an open-circuit voltage was established for 30 min to ensure system stability. The corrosion measurements were then carried out at ± 50 mV around the resting potential at a scan rate of 0.25 mV/s. To define the corrosion current and corrosion potential, the linear sections of the logarithmic current–potential curve were extended with Tafel fittings in Origin 2022 Professional SR1 (Origin Lab, Northampton, MA, USA), and the intersection point was determined. The corrosion rate was calculated using Faraday’s law according to the equation below [52]:

(1) $$R_{m}=\frac{M}{nF\rho }i_{corr}$$

Here, *M* is atomic mass, *n* is the number of electrons involved in the reaction, ρ is density, and *F* is 96,485 C/mol.

### 2.4. Statistics

All calculations and designs of diagrams were executed with Origin 2022 Professional SR1 (OriginLab, Northampton, MA, USA). The conducted measurements are presented by their means and standard deviations. The level of significance was set at *p* < 0.05.

## 3. Results

### 3.1. Tensile Testing

The ultimate tensile strength and the yield strength of ZnAg3 could be determined at 237.5 ± 2.12 MPa and 168 ± 1.41 MPa, respectively. The elongation at fracture was found to be 38.5 ± 1.41%.

### 3.2. Shear Testing

All specimens could be implanted into the artificial bones safely and without distortion.

#### 3.2.1. Fatigue Testing

**Figure 1:**
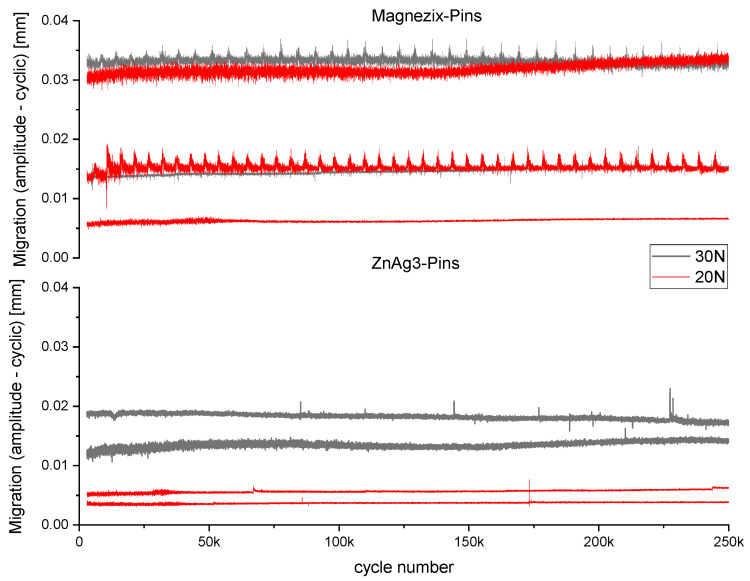
Cyclic loading curve showing the migration (mm) over 250,000 cycles for ZnAg3 pins (bottom) and MAGNEZIX^®^ pins (top) at 30 N (gray) and 20 N (red).

No pin failure with a displacement of >2 mm occurred in all conducted runs. All specimens revealed microscopic relative movements with an amplitude of 5–30 µm, which were similar for both MAGNEZIX^®^ pins and ZnAg3 pins. Over the course of 250,000 cycles, the overall migration did not exceed 100 µm (Figure 1).

#### 3.2.2. Quasi-Static Testing

**Figure 2:**
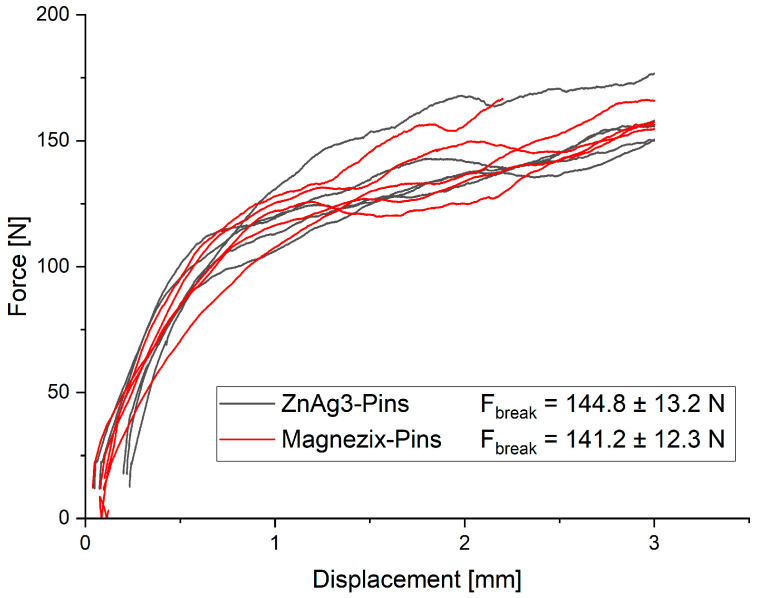
Force–displacement curve for ZnAg3 pins and MAGNEZIX^®^ pins no significant difference (*p* = 0.71484).

The measurements of both pins showed very similar results, with an ultimate load of 144.8 ± 13.2 N for ZnAg3 pins and 141.2 ± 12.3 N for MAGNEZIX^®^ pins (Figure 2). No significant difference could be observed (*p* = 0.71484) between the ZnAg3 pins and MAGNEZIX^®^ pins.

### 3.3. Electrochemical Testing

**Figure 3:**
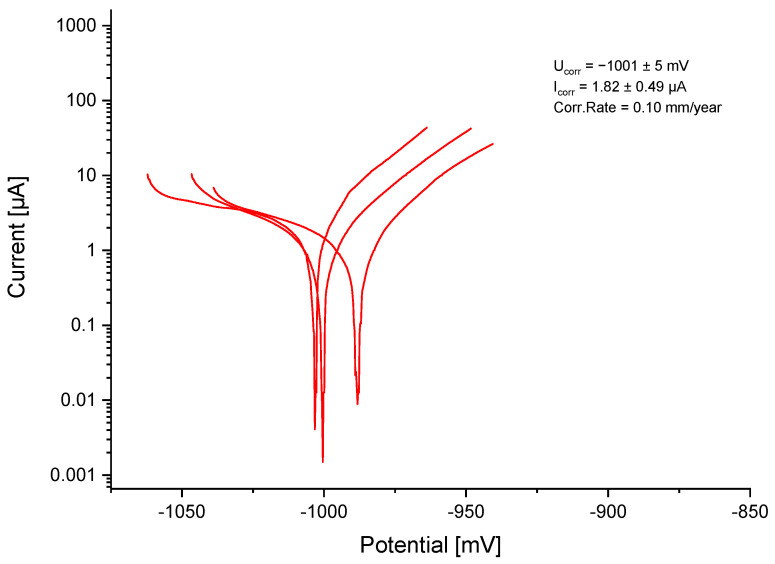
Logarithmic current–potential curve for ZnAg3.

The established OCP was at −1013 ± 28.5 mV. With a corrosion potential (U_corr_) of −1001 ± 5 mV and a corrosion current (I_corr_) of 1.82 ± 0.49 µA, the calculated corrosion rate was 0.10 mm/year for ZnAg3 (Figure 3).

## 4. Discussion

### 4.1. Mechanical Measurements

The results of the tensile testing could display that ZnAg3 showed promising mechanical properties as a bioabsorbable material with an ultimate tensile strength of 237.5 ± 2.12 MPa, a yield strength of 168 ± 1.41 MPa, and an elongation at fracture of 38.5 ± 1.41%. The difficulty in finding suitable materials for bioabsorbable implants is that sufficient strength must be accompanied by high ductility [30,53]. Many materials could not offer both properties. Among others, Mg alloys showed great tensile strength but were poorly ductile, which can cause long-term failure [54,55,56,57]. The tested alloy offered the required ductility with an elongation at fracture of >30% as well as an adequate tensile strength of >200 MPa. In comparison, the MAGNEZIX^®^ pins provide a high tensile strength of >290 MPa and yield strength of >260 MPa, but they do not retain enough ductility with an elongation at fracture of >8% [58].

A significant enhancement in tensile strength while sustaining high elongation at fracture is most likely facilitated by the countervailing effects of precipitation hardening and favorable texture orientation during extrusion [30]. Therefore, the extrusion process and the associated effects on grain size have an impact on the material properties. In this study, ZnAg3 manufactured by Limedion and Quadralux (Limedion GmbH, Mannheim, Germany; Quadralux e.K., Mannheim, Germany) showed a grain size of 5–10 µm after hot extrusion at 250–300 °C with an extrusion rate of 25:1 [47]. In comparison, Sikora-Jasinska et al. [30] reported a UTS of 203 MPa, a YS of 157 MPa, and an elongation at fracture of approximately 34% for ZnAg2.5 with a grain size of 16 µm after hot extrusion at 250 °C with an extrusion rate of 14:1. Furthermore, Yang et al. [59] reported a UTS of 234 MPa, a YS of 188 MPa, and an elongation at fracture of 36% for ZnAg2 with a grain size of <10 µm after hot extrusion at 260 °C with an extrusion rate of 36:1.

It is important to note that, e.g., Bowen et al. [60] proposed different mechanical requirements for bioabsorbable implants such as stents. Here, a tensile strength of >300 MPa, a yield strength of >200 MPa, and an elongation at fracture of >18% were suggested [60]. Although these values are mostly accepted by the research community, it is crucial to notice that they were assimilated from the parameters of permanent 316 L stainless steel (SS) stents [61,62]. A permanent implant is requested to last for a much longer period of time than a bioabsorbable implant. By degrading the implant material and rebuilding the bone structure, the body is able to increasingly take on mechanical stress. As a result, the suggested values may be appropriate for permanent implants but might not comply with the optimum for bioabsorbable implants. In addition, similar mechanical values are being aimed at for different bioabsorbable implant materials, regardless of their usage site. Consequently, identical mechanical requirements are applied to vascular stent devices and bone implant materials, which are not in accordance with the distinct requirements established by their respective applications [39]. Thus, it may be necessary to revise the proposed values in order to distinguish between permanent and bioresorbable implants and to modify the requirements for mechanical performance according to the particular application.

The yield strength indicates the stress that a material can withstand until permanent deformation occurs. As soon as this load is exceeded, the material does not return to its original shape. Most commonly, single-loading event scenarios are performed to determine the load to the point of plastic deformation. In this study, ZnAg3 showed a yield strength of 168 ± 1.41 MPa, which is in the range of results from previously conducted studies [30,38,59]. However, a single-loading event is usually not the reason for implant breakage in clinical use. Typically, cyclic events cause implant failure since repetitive loading especially afflicts weak points, such as the material close to the fracture location. It is known that the yield strength correlates with the number of cycles until failure or breakage of the material in cyclic loading scenarios. This reflects the importance of fatigue testing, which should be carried out as part of the mechanical testing.

To assess a reliable fatigue strength, the experiment should conform to the expected load scenarios. During operations in trauma surgery, two or more bone fragments are usually fixed together. In order to avoid the fragments from sliding against each other, the implant usually has to withstand in particular shear, bending, and torsion forces after surgery [63]. Hence, the testing of orthopedic implant materials should focus on their shear, bending, and torsion fatigue strengths [29]. For this reason, the tests conducted included quasi-static shear testing, mimicking the mechanical stress after hallux-valgus surgery.

The results of the shear testing could prove sufficient fatigue strength as well as adequate resistance during the quasi-static testing of ZnAg3. The tested alloy could withstand the expected load after hallux-valgus surgery with approximately 5000 to 7000 steps a day, with a maximum load of 30 N per step for a period of 4 to 6 weeks until the healing process is expected to be concluded [48,64]. Here, the performance of ZnAg3 pins was equal to the MAGNEZIX^®^ pins, which corresponds to the data in previous studies [65]. Moreover, a shear strength of 144.8 ± 13.2 N for the ZnAg3 pins qualifies the tested alloy as a fixation device in orthopedics and trauma surgery since there was no significant difference from the MAGNEZIX^®^ pins with a shear strength of 141.2 ± 12.3 N, which are already in clinical use.

The shear tests were conducted in air and thus did not simulate physiological conditions, since the material is surrounded by body fluid in clinical use. Here, degradation of the implant material would take place, and might ultimately affect the mechanical properties. Very few studies have investigated this degradation process and its effects on zinc alloys. For example, Li et al. [66] examined the tensile strength of pure zinc and zinc alloys containing 1 wt% of magnesium, calcium, and strontium, respectively, after 8 weeks of immersion in simulated body fluid (m-SBF). The results showed that zinc and its alloys are able to maintain their mechanical integrity. Törne et al. [67] could prove that pure zinc in simulated body fluid would continue to show high ductility and great resistance to stress corrosion cracking. According to these results, it is very likely that the tested alloy in this study maintains its mechanical properties in a similar manner. This implies that the outcomes achieved are highly likely to be applicable to actual conditions.

When performing shear tests, such as in this study, the used bone substitute material and its holding forces play an important role. The 20 PCF cellular rigid polyurethane foam used in this study resembled the average density of a metatarsal bone at 0.32 g/cm^3^ [68]. This ensures a high degree of comparability with physiological conditions. For instance, Seebeck et al. [69] utilized human tibiae in their research and achieved significantly higher values compared to studies that used synthetic bone [65]. However, since there is a high degree of variation in bone structure, bone density, and bone composition when using cadaver bones, synthetic bone should be preferred in experimental tests in the interest of reproducibility [70].

### 4.2. Electrochemical Measurements

The corrosion rate of the tested ZnAg3 alloy was, at 0.10 mm/year, in the expected range between 0.05 and 0.15 mm/year, according to previous studies [17,38]. This could also prove that ZnAg3 showed a much higher corrosion resistance than Mg alloys and thus would provide better long-term stability in clinical use [55,71]. Hagelstein et al. [38] described a corrosion rate of 0.14 mm/year for ZnAg3 with an Ag content of 3.0 wt% manufactured by Limedion and Quadralux (Limedion GmbH, Mannheim, Germany; Quadralux e.K., Mannheim, Germany), which is slightly higher than the measured corrosion rate in this study. This circumstance might be due to the fact that the grain size of the ZnAg3 in this study was determined to be smaller at 5–10 µm and the Ag content was higher at 3.3 wt%, as previous investigations could show [47]. Thus, it is apparent that the grain size in fact impacts the corrosion rate. A homogeneous material with a small grain size automatically offers a significantly smoother surface, which is likely to increase the corrosion resistance. Salahshoor et al. [72] showed that a decrease in the corrosion rate of up to 50% is possible by altering the grain size through different manufacturing techniques in Mg–Ca alloys.

For bioabsorbable implants, the suggested values vary greatly and are highly dependent on the implantation site. Bowen et al. [28] proposed a required corrosion rate of 0.02 mm/year for biodegradable stent material. In comparison, e.g., Venezuela et al. [18] mentioned in their review a corrosion rate of approximately 0.04 mm/year to 0.14 mm/year for fixation devices such as screws or plates. With a degradation rate of 0.05–0.15 mm/year, the investigated ZnAg3 alloy is therefore within the range suggested by Venezuela et al. [18]. Overall, a bioabsorbable material in orthopedics and trauma surgery is requested to sustain its mechanical integrity for at least 3–6 months and subsequently dissolve completely within 1–2 years [59]. Yang et al. [59] could display that the degradation rate of pure zinc is too low, at approximately 0.015 mm/year. Moreover, the in vitro assessment was matched with in vivo observations regarding the volume loss. Here, pure zinc showed a volume loss of less than 10% after 8 weeks, which reflects an inadequately low corrosion rate.

A reasonable corrosion rate should be assured before considering a bioabsorbable material for clinical use. The measurements were conducted in an artificial environment, which does not fully represent physiological conditions. In order to be able to estimate the actual corrosion resistance of the tested alloy, referring to previous in vivo studies can be of avail. Yang et al. [59] introduced a ZnAg2 alloy into the rat model and could observe accelerated degradation compared to in vitro measurements. This finding seems reasonable since the implant material in vivo is subjected to the stress-shielding response of the organism after implantation [45]. That suggests that the corrosion behavior of ZnAg3 would similarly be reflected in living organisms, which would be highly compatible with bone formation at the measured corrosion rate, allowing sufficient osteointegration. Nonetheless, the performance of ZnAg3 as a bioabsorbable material needs to be confirmed with in vivo studies before considering clinical use.

## 5. Conclusions

The mechanical properties of ZnAg3 meet the requirements for bioabsorbable materials, as evidenced by the measurements conducted in this study. As requested for osteosynthetic implants, ZnAg3 offers a considerable tensile strength with high ductility as well as great shear strength, providing the needed mechanical stability during bone healing processes. An appropriate corrosion rate ensures adequate durability throughout the period of bone healing, thereby enabling the bone to form new bone material while still retaining sufficient stability. The results of this study are consistent with previous findings and therefore support the assumption that zinc–silver alloys are very suitable candidates for further investigations. Due to its beneficial mechanical properties and its distinguished biocompatibility, ZnAg3 represents a promising choice for in vivo studies.

## 6. Patents

The alloy, including its casting and extrusion process, is patented by the company Limedion. It includes the production of zinc with 90.0–99.5 wt% and silver with 0.05–10 wt%. The patent specification number is EP 3 250 247 B1.

## Data Availability

The data presented in this study are available on request from the corresponding author.
